# DDIT3 Promotes Starvation-Induced Autophagy via ER Stress in Vero Cells

**DOI:** 10.3390/ijms27104315

**Published:** 2026-05-12

**Authors:** Muzi Li, Renhou Jia, Rong Huang, Jiamin Wang, Zilin Qiao, Na Sun

**Affiliations:** 1 Ministry of Education Engineering Research Centre for Key Technology and Industrialisation of Cell-Based Vaccines, Northwest Minzu University, Lanzhou 730030, China; limuzi61@163.com (M.L.); 18009485046@163.com (R.J.); huangrong78423@163.com (R.H.); jiaminwang1987@163.com (J.W.); qiaozilin@xbmu.edu.cn (Z.Q.); 2 Gansu Tech Innovation Center of Animal Cell, Biomedical Research Center, Northwest Minzu University, Lanzhou 730030, China; 3 Key Laboratory of Biotechnology and Bioengineering of State Ethnic Affairs Commission, Biomedical Research Center, Northwest Minzu University, Lanzhou 730030, China; 4 Gansu Provincial Bioengineering Materials Engineering Research Center, Lanzhou 730010, China

**Keywords:** Vero cells, *DDIT3*, autophagy, endoplasmic reticulum stress (Ers)

## Abstract

Vero cells in high-density vaccine cultures often face nutrient starvation, especially in suspension-adapted Vero cells. Previous studies showed that serum starvation dramatically enhances autophagy and mitophagy in suspension-adapted Vero cells. Transcriptomic profiling also revealed significant upregulation of *DDIT3*, a marker of endoplasmic reticulum stress (ERS), in suspension-adapted Vero cells compared to adherent cells. To investigate the functional role of *DDIT3*, an Earle’s Balanced Salt Solution (EBSS)-induced starvation model was established in adherent Vero cells, recapitulating key autophagy and ER stress responses observed under suspension conditions. The genetic silencing of *DDIT3* by shRNA attenuated autophagy, as evidenced by a reduced LC3-II/LC3-I ratio and impaired autophagosome–lysosome activity. Notably, *DDIT3* knockdown enhanced cell proliferation and increased the yield of H1N1 influenza virus under nutrient-deprived conditions. Collectively, these results suggest that DDIT3 may serve as a critical regulator linking ER stress to autophagy in Vero cells, and that the suppression of DDIT3 may represent a promising strategy for developing autophagy-resistant Vero cell lines suitable for high-density suspension culture in vaccine production.

## 1. Introduction

The Vero cell line was derived from the kidneys of the African green monkey and is an adherent epithelial cell line. Vero cells serve as an important component in the pro-duction of human vaccines and have been approved by the World Health Organization (WHO) for this specific purpose [1]. The unique attributes of the Vero cell line, including broad viral susceptibility, an inherent inability to secrete interferon, and non-tumorigenicity, make this an exceptionally valuable substrate for the production of vaccines against viruses such as rabies, polio, and SARS-CoV-2 [2,3]. The vaccine production industry is now adopting scalable suspension culture processes for higher yield to meet global vaccine demand. Nonetheless, a major bottleneck remains in that it is difficult for Vero cells to adapt to high-density culture in serum-free suspension. The sticky properties and serum dependency of Vero cells significantly limit their application in large-scale bioproduction [4].

Withdrawing serum, a complex source of growth factors and nutrients, is a critical step in the adaptation of cells. Placing cells in serum-free media causes severe metabolic stress, the loss of proteostasis, and endoplasmic reticulum stress (ERS). This can hyperactivate the pro-death unfolded protein response (UPR) and the catabolic process of autophagy [5]. Normally, autophagy represents a pro-survival process; however, upon prolonged stress, autophagy becomes excessive and leads to the degradation of essential cellular components, eventually leading to growth arrest and cell death [6]. This type of cell death is a major physiological barrier that is responsible for limiting the growth of Vero cells to the high densities required for manufacturing vaccines on an industrial scale. For this reason, it is crucial to understand and control the signaling pathways that couple serum starvation to autophagic activation if we are to develop suspension-adapted Vero cell lines.

Megan et al. cultured Vero cells with serum-free CDM2 and then performed transcriptomic analyses to compare differentially expressed genes between suspended and adherent Vero cells. These authors showed that suspended Vero cells feature abnormalities in materials, energy metabolism, and DNA replication, along with more extensive autophagy [7]. However, researchers have yet to optimize the autophagy of suspended Vero cells. Previously, we domesticated Vero cells using low-serum Vero-S and used RNA-seq analysis to prove that the levels of autophagy were extremely significant. Following the gradual reduction of serum concentration in suspensions of Vero cells during adaptation, we observed intense autophagy and the impairment of cell viability, thus preventing the attainment of a high cell density. In another study, Baek et al. showed that 3-MA suppressed autophagy during the production of a monoclonal antibody, considerably enhancing the density of viable cells and the expression of recombinant protein [8]. The recent use of RNAi to knock down Aven, a regulator of autophagy, has generated starvation-resistant cells; engineered cells lived for three days longer than their parent cells. Furthermore, these cells achieved a peak density of 4.7 × 10^6^ cells/mL with >90% viability. Overall, this research has led to significant enhancements in cell yield and protein production [9]. Consequently, it is clear that autophagy is an essential factor that determines the large-scale culture of Vero cells because it regulates both cell death and proliferation. Improving culture performance entails the specific reduction of autophagy activity to promote continuous cell growth [10]. In addition, previous studies have reported ER stress to be involved in the induction of autophagy by nutrient deprivation, although specific details remain unknown.

The ER can sense nutrient fluxes and metabolic stress. Serum starvation is known to alter homeostasis in the ER, leading to an accumulation of unfolded proteins that activate the UPR via PERK, IRE1α, and ATF6 [11]. The transcription factor DDIT3 (also referred to as CHOP) is an important downstream effector of the UPR [12]. Although DDIT3 (DNA-damage-inducible transcript 3) has long been implicated in mediating apoptosis, recent findings indicate that this protein may also promote autophagy as well [13]. No previous research study has investigated the specific function of the ER_S_-DDIT3 pathway in mediating the autophagic response to serum starvation in Vero cells. For example, it is possible that DDIT3 might drive the excessive autophagy that limits the density of Vero cells in culture. Furthermore, it might be possible to target this pathway to stimulate cell survival and growth under nutrient stress.

To elucidate how serum starvation inhibits the proliferation of suspended Vero cells, we performed RNA-seq-based transcriptomic analysis to systematically identify dysregulated autophagy-related genes and delineate the link to ERS-responsive autophagy. Based on this profiling, we identified DDIT3 as a downstream target of the UPR activation pathway. Subsequently, we established DDIT3 knockdown cell lines using lentiviral transfection technology to elucidate the specific mechanism responsible for the ability of DDIT3 to regulate autophagy in Vero cells and its relationship with the ER. We also determined the proliferation and susceptibility to influenza A virus in sh-DDIT3 Vero cells. Collectively, our findings provide preliminary evidence to support the fact that targeting DDIT3 may enhance the starvation tolerance of Vero cells, thus offering a potential target to improve the efficiency and stability of adaptation to serum-free suspension.

## 2. Results

### 2.1. Starvation-Induced Autophagy in Vero Cells

**Figure 1:**
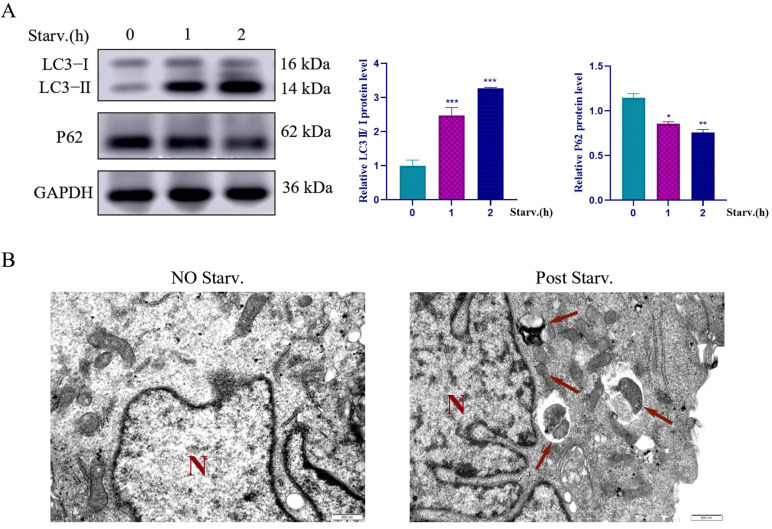
Nutrient deprivation induces autophagy in Vero cells. Vero cells were subjected to EBSS starvation for 0, 1, or 2 h, followed by the detection of autophagy-related protein expression levels and subcellular structural changes. (**A**) Western blotting analysis of the autophagy markers LC3-I, LC3-II, and p62 protein expression levels, with GAPDH used as a loading control. Representative immunoblots are shown on the left, with quantitative analysis on the right. (**B**) Transmission electron microscopy observation of Vero cell ultrastructure. (**Left** panel): under normal culture conditions (non-starved). (**Right** panel): after starvation treatment, numerous autolysosomes were observed (indicated by red arrows), some of which contained engulfed mitochondrial fragments. Magnification: ×25,000. Abbreviations: N, nucleus. To observe the formation of autophagy bodies in Vero cells under starvation-induced condition (red arrows indicate autophagy bodies; N indicates the cell nucleus; ×25,000). * *p* < 0.05, ** *p* < 0.01, *** *p* < 0.001.

Based on prior studies of Vero suspension and adherent cells, it has been confirmed that Vero suspension cells exhibit an autophagy-inducing microenvironment due to nutrient deprivation. However, suspension cells are challenging to manipulate experimentally. Therefore, we employed adherent cells to establish a starvation model analogous to that in suspension cells. To confirm that nutrient deprivation increases autophagy in Vero cells, we first examined the autophagy markers LC3 and p62 by Western blotting. When compared to control cultures, 1–2 h starved cells show a major and significant increase in the level of LC3-II/I and a significant decrease in p62 levels (Figure 1A). Ultrastructural validation by the TEM analysis confirmed that control cells maintain intact mitochondria and well-ordered rough endoplasmic reticulum. On the other hand, starved cells exhibited mildly condensed mitochondria and a large number of autolysosomes. Most of these autolysosomes contain an engulfed mitochondrial fragment (Figure 1B). These data strongly show that nutrient deprivation induces autophagy in Vero cells.

### 2.2. RNA Expression Profiling Analysis in Starved Vero Cells

**Figure 2:**
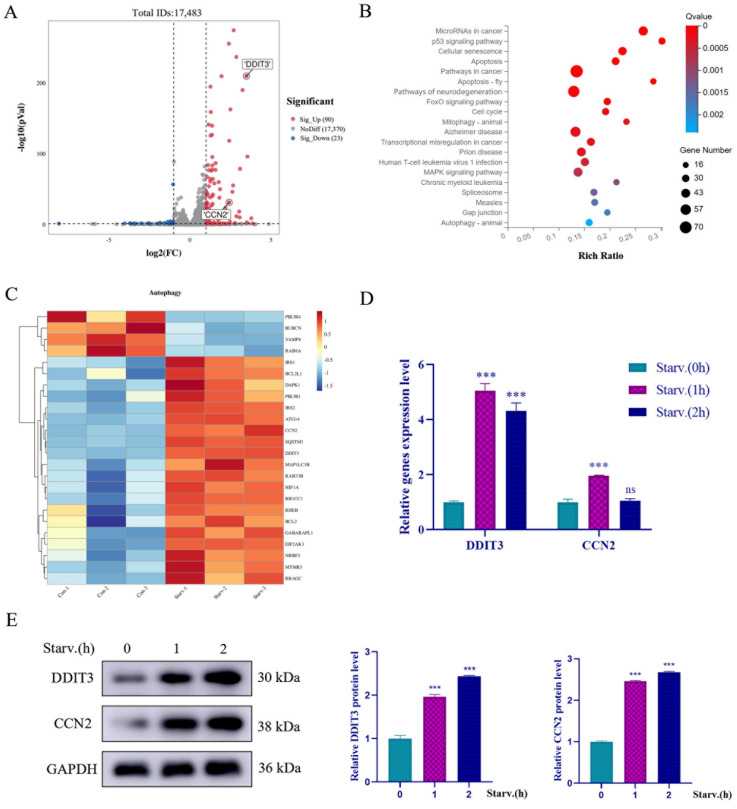
RNA expression profiling of Vero cells under nutrient deprivation. After starving Vero cells for 2 h, total RNA was extracted for transcriptome sequencing and analysis. Differential gene screening criteria: *p* < 0.05 and |FC| > 2. (**A**) Volcano plot of differentially expressed genes identified by high-throughput sequencing. Red dots indicate upregulated genes, gray dots indicate genes with no significant change, and blue dots indicate downregulated genes. (**B**) KEGG pathway enrichment analysis of differentially expressed genes. Bubble size represents the number of genes enriched in each pathway. (**C**) Cluster analysis of differentially expressed genes in autophagy pathways induced by starvation. (**D**) qRT-PCR detection of *DDIT3* and *CCN2* expression in Vero cells, with and without nutrient starvation. *n* = 3; *** *p* < 0.001; ns: *p* > 0.05. (**E**) Western blotting detection of *DDIT3* and *CCN2* expression in Vero cells, with and without nutrient starvation. *n* = 3; *** *p* < 0.001.

To identify the key genetic determinants of the responses of Vero cells to stress under nutrient deprivation, we performed high-depth RNA-seq on control and starved cultures and mapped 17,483 expressed transcripts. Starvation caused a significant difference in gene expression between the two groups; 90 were upregulated and 23 were downregulated. Figure 2A shows the 113 differentially expressed genes. Results from KEGG pathway enrichment (*q* < 0.05) indicated marked activation of general autophagy and mitophagy, together with activation of apoptosis and the p53 stress-response network (Figure 2B). Consequently, we can conclude that autophagy is the first main cellular adaptive response employed by these Vero cells. We screened DEGs with |log_2_FC| > 1 and then produced a clustered heatmap of the 24 autophagy-associated genes. Core autophagy modulators ATG14, VAMP8, and MAP1LC3B were robustly induced. It is noteworthy that *CCN2* and *DDIT3*, two well-known regulators of endoplasmic reticulum (ER) stress, were found to be highly upregulated in starved Vero cells (Figure 2A,D). We thus hypothesized that these factors play a pivotal role in starvation-induced autophagy. RT-qPCR data confirmed strong, starvation-dependent induction of *DDIT3* and *CCN2* mRNA. Western blot analysis corroborated these findings, revealing substantial elevations of *DDIT3* and *CCN2* at the protein level at 1–2 h post nutrient deprivation (Figure 2E). Across the assays, *DDIT3* showed a more robust quantitative response than *CCN2*. Based on this stronger and more consistent activation, *DDIT3* was chosen for subsequent mechanistic studies aimed at understanding autophagy regulation during nutrient deprivation.

### 2.3. Knockdown of DDIT3 Reduces Starvation-Induced Autophagy in Vero Cells

**Figure 3:**
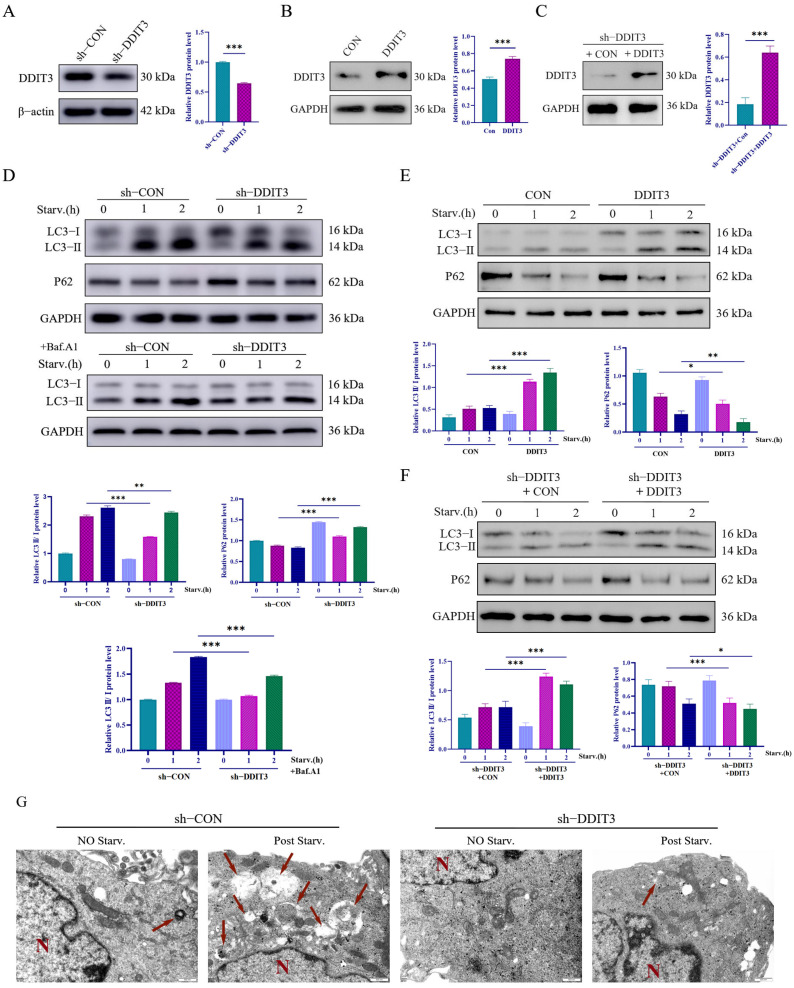
*DDIT3* upregulation is required for autophagy induction. (**A**–**C**) Western blotting detection of *DDIT3* expression in stable cell lines. *n* = 3; *** *p* < 0.001. (**D**) Analyzing LC3II levels in the presence or absence of the lysosomal inhibitor bafilomycin A1 (Baf.A1) revealed that autophagy flux is impaired when *DDIT3* expression is silenced. As another indicator of autophagy, p62 is an autophagosome cargo protein that degrades in autolysosomes. While nutrient starvation-induced p62 degradation in control cells, *DDIT3* ablation resulted in p62 accumulation. (**E**,**F**) Western blotting detection of LAMP1 and TFEB expression in stable cell lines, with and without nutrient starvation. *n* = 3; * *p* < 0.05, ** *p* < 0.01, *** *p* < 0.001. (**G**) Transmission electron microscopy observation of autophagosome formation in *DDIT3* stably knocked-down cell lines (red arrows indicate autophagy bodies; N indicates the cell nucleus; ×25,000).

**Figure A1:**
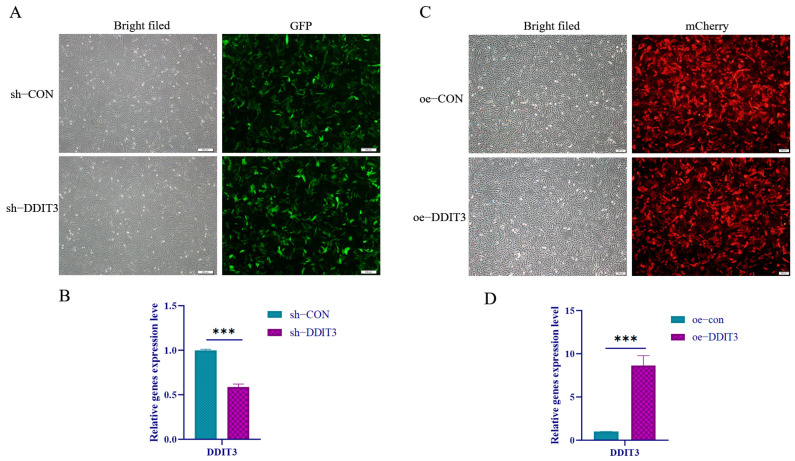
Knockdown and overexpression efficiency of DDIT3 in Vero cells. (**A**,**C**) GFP and mCherry fluorescence expression of stable cell line observed by fluorescence microscope, Scale bars, 200 μm. (**B**,**D**) qRT-PCR detection of DDIT3 expression in stable cell lines. *n* = 3; *** *p* < 0.001.

To find out if the induction of DDIT3 has a mechanistic connection with autophagy triggered by starvation, we generated stable DDIT3 knockdown and DDIT3-overexpressing Vero cells by lentivirus shRNA. Puromycin (4 µg mL^−1^, 72 h) and neomycin (800 µg mL^−1^, 72 h) selections yielded > 80% GFP- and mCherry-positive cells, respectively (Figure A1A,C). Analysis using RT-qPCR showed a significant decrease in DDIT3 mRNA levels compared to sh-CON control (Figure A1B), while Western blot indicated a drop in DDIT3 protein levels (Figure 3A). The results show that both transcription and translation of *DDIT3* were downregulated, indicating that a *DDIT3*-deficient Vero line could be developed.

To investigate the functional role of *DDIT3* in starvation-induced autophagy, the autophagy markers LC3 and p62 were examined in sh-*DDIT3* and sh-CON Vero cells subjected to starvation. In both sh-CON and sh-*DDIT3* cells, starvation for 1–2 h triggered the expected autophagy signature: an increase in the LC3-II/I ratio and the decline of p62. Under starvation, the sh-*DDIT3* cells showed a significantly lower LC3-II/I ratio and a higher residual p62 level than the sh-CON cells, indicating attenuated autophagy (Figure 3D). We tested Baf A1 in order to quantify the accumulation of autophagosomes. By starving the cells again, the authors were able to see an elevation of LC3-II once again in sh-*DDIT3* cells, but the increase was much less than in sh-CON cells. This means that *DDIT3* depletion represses autophagosome formation (Figure 3D). In further ultrastructural analysis, non-starved cells of both genotypes showed intact mitochondria and rough ER. Following starvation, sh-CON cells showed significant shrinkage of mitochondria and ER and abundant autolysosomes. On the other hand, sh-*DDIT3* cells exhibited only minor mitochondrial condensation, as well as very low numbers of autolysosomes, while the rough ER remained largely intact (Figure 3G). Together, these data showed that knocking down *DDIT3* drastically inhibits starvation-induced autophagy in Vero cells with a defect in autophagosome biogenesis and autolysosome formation.

To further validate our mechanistic conclusions, gain-of-function experiments were conducted by overexpressing DDIT3 in Vero cells. Western blot analysis confirmed a marked increase in DDIT3 protein levels following transfection with a DDIT3 overexpression plasmid (Figure 3B). Under EBSS-induced starvation conditions, DDIT3-overexpressing cells exhibited a higher LC3-II/I ratio and enhanced p62 degradation, indicating increased autophagy flux (Figure 3E). In parallel, rescue experiments were performed on sh-DDIT3 knockdown cell lines. Reintroduction of DDIT3 in these cells similarly restored DDIT3 protein expression and recapitulated the autophagic phenotype, with an elevated LC3-II/I ratio and accelerated p62 degradation under starvation (Figure 3C,F). These gain-of-function results are consistent with the findings from knockdown experiments, collectively demonstrating that DDIT3 positively regulates starvation-induced autophagy in Vero cells.

### 2.4. DDIT3 Depletion Impairs Lysosomal Function in Vero Cells

**Figure 4:**
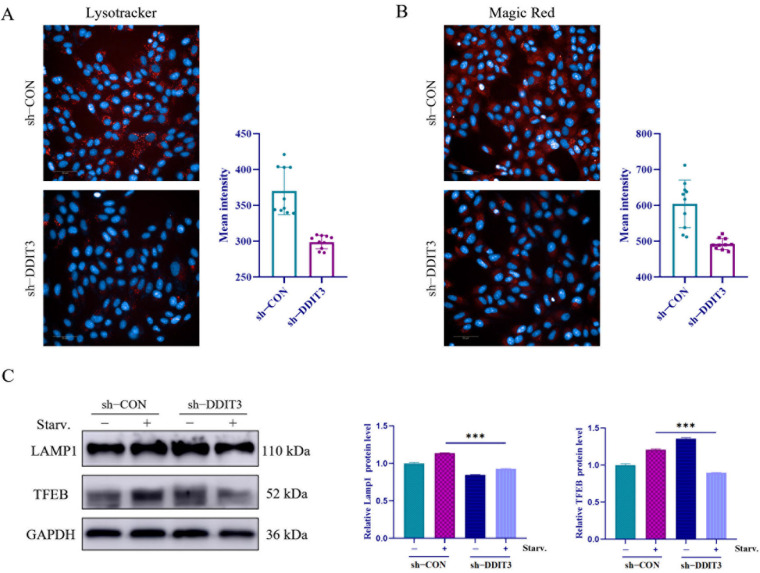
*DDIT3* depletion impairs lysosomal function in Vero cells. Lysosomal acidification, cathepsin B activity, and lysosomal-related protein expression were examined in sh-CON (control knockdown) and sh-DDIT3 (DDIT3 knockdown) Vero cells. (**A**) Detection of lysosomal acidification using LysoTracker Red staining. Representative fluorescence images are shown on the left, with quantitative analysis of fluorescence intensity on the right. Scale bar: 50 μm. (**B**) Detection of cathepsin B activity using Magic Red staining. Representative fluorescence images are shown on the left, with quantitative analysis of fluorescence intensity on the right. Scale bar: 50 μm. (**C**) Western blotting analysis of lysosomal-related protein LAMP1 and TFEB expression levels, with GAPDH used as a loading control. Representative immunoblots are shown on the left, with quantitative analysis on the right. *n* = 3; *** *p* < 0.001.

Lysosomes are the final degrading area of autophagy that fuse with autophagosomes to create autolysosomes to degrade faulty organelles and improper proteins. In order to assess the lysosomal function upon *DDIT3* knockdown, sh-*DDIT3* and sh-CON Vero cells were stained with LysoTracker Red. As compared to controls, sh-*DDIT3* cells exhibited markedly dimmer red fluorescence (Figure 4A) because LysoTracker Red accumulates in proportion to acidity. Thus, the dimmer signal indicates a higher lysosomal pH and weaker acidification. The Magic Red labelling of active cathepsin B showed weaker fluorescence in sh-*DDIT3* cells on a regular basis (Figure 4B). To further evaluate the impact of DDIT3 knockdown on lysosomal function, the protein expression levels of LAMP1 and TFEB were examined by Western blotting. The results showed that in sh-CON control cells, starvation treatment significantly induced the upregulation of LAMP1 and TFEB expression. In contrast, in sh-DDIT3 cells, the starvation-induced expression of LAMP1 and TFEB was markedly reduced compared to sh-CON cells (Figure 4C). Thus, DDIT3 knockdown affected lysosomal acidification and cathepsin B activity and disrupted TFEB-mediated lysosome–autophagosome fusion, thus preventing the formation of autolysosomes and starvation-induced autophagy in Vero cells.

### 2.5. ER Stress-Upregulated DDIT3 Promotes Autophagy in Vero Cells

**Figure 5:**
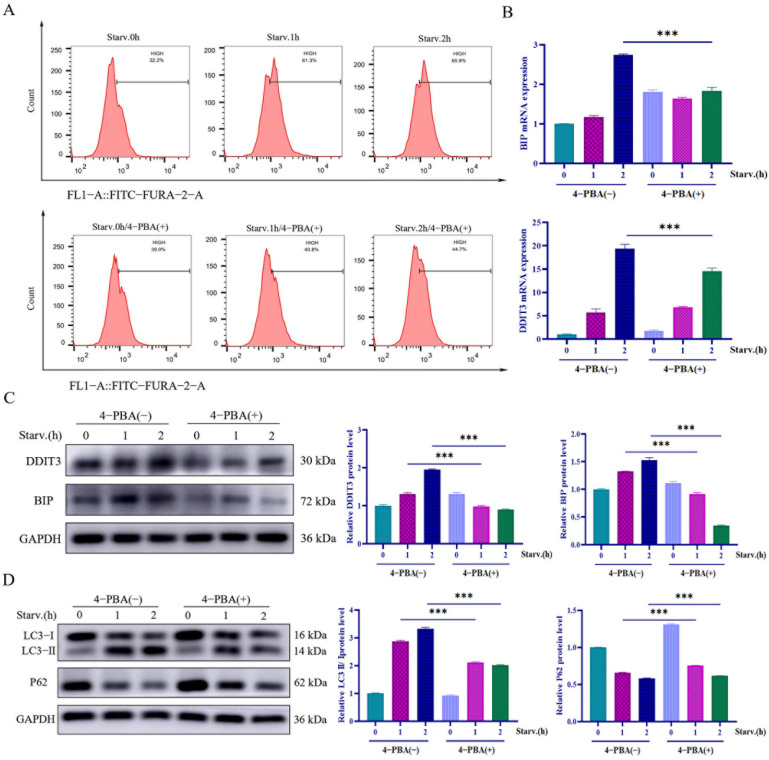
Upregulation of *DDIT3* by ER stress induces autophagy in Vero cells. (**A**) Flow cytometry was used to measure intracellular Ca^2+^ concentration via Fluo-4/AM fluorescent probes. (**B**) qRT-PCR to detect the expression of BIP and *DDIT3* in cells after 4-PBA treatment. *n* = 3; *** *p* < 0.001. (**C**,**D**) Western blotting to detect the expression of BIP, *DDIT3*, LC3, and p62 in cells after 4-PBA treatment. *n* = 3; *** *p* < 0.001.

*DDIT3* plays a critical role as a downstream effector of the endoplasmic reticulum stress response. It regulates autophagy by linking processes in ER stress signaling to cell fate. In order to demonstrate how *DDIT3* controls autophagy, its interplay with ERs needs to be established. The endoplasmic reticulum (ER) is the largest intracellular Ca^2+^ reservoir. Ca^2+^ storage and release are indispensable for physiological homeostasis and signal transduction. Ca^2+^ dyshomeostasis is a hallmark of ERs. We used a Ca^2+^-sensitive fluorescent probe to check intracellular Ca^2+^ dynamics under starvation. As shown in Figure 5A, after 1 h and 2 h of nutrient deprivation, intracellular Ca^2+^ levels increased by 29.1% and 33.7%, respectively, compared to non-starved controls. Upon further addition of the ER stress inhibitor 4-phenylbutyrate (4-PBA), Ca^2+^ signals at the respective time points were reduced by 20.5% and 21.2% (Figure 5A). Thus, starvation time-dependently enhances cytosolic Ca^2+^ fluorescence in Vero cells, indicating progressive ERS activation. We computed the levels of the canonical endoplasmic reticulum stress markers BIP and *DDIT3* as mRNA by RT-qPCR to further ascertain that Vero cells experience endoplasmic reticulum stress upon nutrient deprivation. As depicted in Figure 5B, both transcripts increased dramatically 1–2 h after starvation compared to control cells, and treatment with the ER-stress inhibitor 4-phenylbutyrate (4-PBA) for 2 h completely reversed this increase. As shown by Western blotting analysis, BIP as well as *DDIT3* protein levels rose in concert with their mRNA expression as a function of starvation and were significantly suppressed following exposure to 1–2 h of 4-PBA (Figure 5C). In summary, our results demonstrate that nutrient deprivation strongly triggers ER stress in Vero cells and that *DDIT3* expression is strictly dependent on ER stress signaling.

In order to investigate the functional relationship between ER stress and autophagy, Vero cells treated or untreated with 4-PBA were subjected to a starvation medium, and the markers LC3 and p62 were analyzed by Western blotting. It showed that when fed controls were used, both 4-PBA-treated and untreated cells had increased LC3-II/I ratios and decreased p62 levels after 1–2 h of starvation, indicating autophagy induction. It is worth mentioning that, compared to untreated cells subject to starvation, 4-PBA-treated cells displayed a significantly lower LC3-II/I ratio and a higher p62 level (Figure 5D). The study findings suggest that ERs not only drives DDIT3 expression but also modulates starvation-induced autophagy in Vero cells.

### 2.6. DDIT3 Regulates the AKT–mTOR Signaling Pathway to Affect Autophagy in Vero Cells

**Figure 6:**
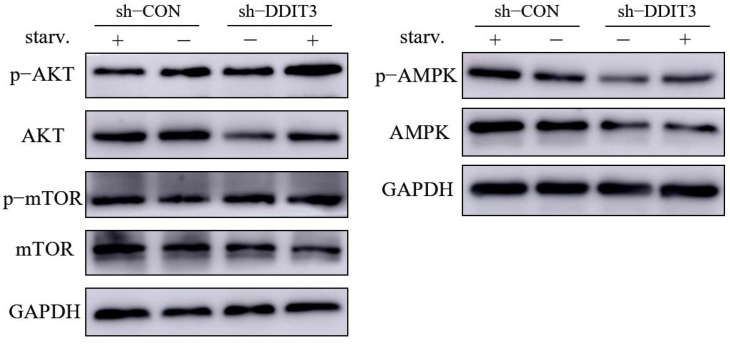
Analysis of DDIT3 regulation of proteins associated with the AKT/mTOR signaling pathway. Western blotting was performed to detect the expression levels of AKT/mTOR and AMPK signaling pathway-related proteins in sh-CON (control knockdown) and sh-DDIT3 (DDIT3 knockdown) Vero cells under normal culture conditions (−) or EBSS starvation treatment (+). The detected markers included p-AKT, total AKT, p-AMPK, total AMPK, p-mTOR, and total mTOR, with GAPDH used as a loading control.

To investigate the signaling pathway through which DDIT3 regulates autophagy in Vero cells, Western blotting was performed to detect the expression of proteins associated with the AKT/mTOR and AMPK signaling pathways. Results are shown in Figure 6. Compared to non-starved cells, starvation-induced p-AKT/AKT expression was significantly downregulated in the sh-CON control group, while p-AMPK/AMPK expression was significantly upregulated. These findings indicate that under starvation conditions, the AKT/mTOR pathway is suppressed, while the AMPK pathway is activated, which is consistent with the established mechanism of starvation-induced autophagy. Furthermore, compared to sh-CON cells, DDIT3 knockdown cells exhibited significantly increased p-AKT/AKT and p-mTOR/mTOR expression under nutrient deprivation, while p-AMPK/AMPK expression showed no significant change. This suggests that DDIT3 knockdown relieves the starvation-induced suppression of the AKT/mTOR pathway, leading to its activation and subsequent inhibition of autophagy. Taken together, these results indicate that DDIT3 influences autophagy in Vero cells by regulating the phosphorylation and expression of proteins associated with the AKT/mTOR signaling pathway.

### 2.7. DDIT3 Affects the Growth of Vero Cells Under Starvation Conditions

**Figure 7:**
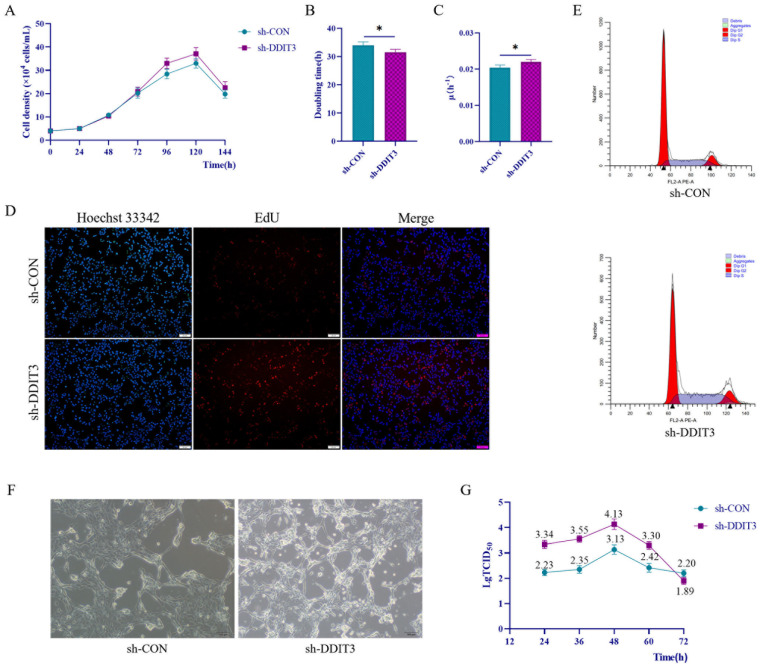
Growth characteristics of sh-CON cells and sh-*DDIT3* cells. (**A**) Growth curves. (**B**) Doubling time. (**C**) Specific growth rate. *n* = 3; * *p* < 0.05. (**D**) Proliferation of sh-CON cells and sh-*DDIT3* cells detected by EdU, Scale bars, 50 μm. (**E**) Cell cycle of sh-CON cells and sh-*DDIT3* cells detected by flow cytometry. (**F**) Proliferation of H1N1 on sh-CON cells and sh-*DDIT3* cells, Scale bars, 100 μm. (**G**) LgTCID50 at different times.

Vero cells have a broad virus range and are widely used for vaccine manufacture; thus, their growth rate and virus susceptibility are vital parameters in biopharmaceutical production. To evaluate the influence of *DDIT3* expression on Vero cell proliferation, sh-*DDIT3* and sh-CON cells were cultured in nutrient-deficient media, and growth was recorded every 24 h. Standard growth curves were generated by fitting the average cell density. As observed in Figure 7A, sh-*DDIT3* cells proliferated significantly faster than sh-CON cells. Analysis through quantitative methods showed doubling time estimated at 31.56 h and 33.99 h. Moreover, the specific growth rate was estimated at 0.022 h^−1^ and 0.020 h^−1^ in sh-*DDIT3* and sh-CON cells, respectively (Figure 7B,C). *DDIT3* depletion caused a 10% increase in specific growth rate under limited conditions. To support these findings, we conducted EdU incorporation assays for DNA synthesis. In line with the growth curve information, we observed a strong increase in EdU-positive nuclei in the sh-*DDIT3* cells, with intense red staining, in comparison to the control (Figure 7D). The cell cycle distribution was analyzed using flow cytometry to confirm the signaling advantage due to *DDIT3* knockdown. As illustrated in Figure 7E, the S-phase fraction was enhanced from 26.52% in sh-CON cells to 39.14% in sh-*DDIT3* cells, indicating that silencing *DDIT3* promotes S-phase entry and accelerates cell cycle progression under nutrient-deprived conditions.

**Figure A2:**
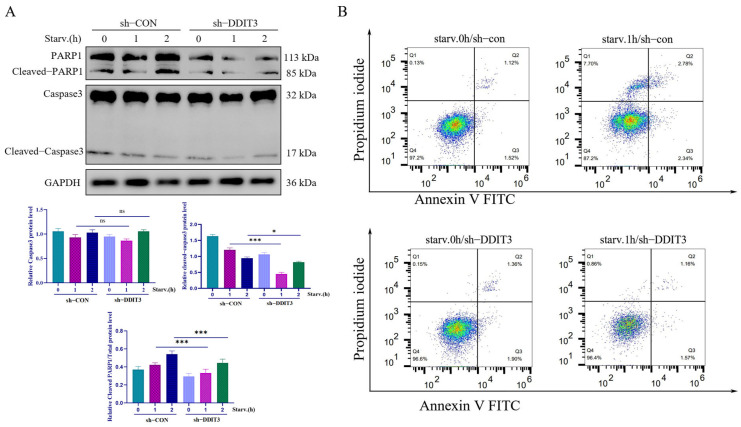
(**A**) Western blotting detection of PARP1 and caspase 3 expression in stable cell lines with and without nutrient starvation. *n* = 3; * *p* < 0.05, *** *p* < 0.001. (**B**) Flow cytometric detection of apoptosis in sh-con and sh-DDIT3 Vero cells with and without nutrient starvation.

To investigate the potential role of DDIT3 in the regulation of apoptosis, we assessed starvation-induced apoptotic responses. Western blot analysis revealed no significant changes in the protein levels of full-length caspase-3 under starvation conditions (Figure A2A). However, upon DDIT3 knockdown, the levels of cleaved caspase-3 and cleaved PARP1 were markedly reduced, suggesting that DDIT3 depletion partially attenuates starvation-induced apoptosis. Flow cytometry analysis (Figure A2B) further demonstrated that DDIT3 knockdown moderately improved the apoptotic status of Vero cells under starvation conditions. Collectively, these results indicate that DDIT3 knockdown exerts a modest protective effect against starvation-induced apoptosis without affecting cell proliferation.

We analyzed the effect of *DDIT3* on H1N1 influenza virus replication by determining viral titers in sh-*DDIT3* and sh-CON Vero cells via TCID_50_ assay. As depicted in Figure 7, we observed a standard kinetics profile, with a rise and fall of viral loads in both groups over 24–72 h post infection, as shown in Figure 7F,G. Notably, sh-*DDIT3* cells consistently showed much higher viral titers than sh-CON cells from 24 h to 60 h. Overall, the data show that depleting *DDIT3* significantly increases the replication of H1N1 in Vero cells.

## 3. Discussion

During the large-scale manufacturing of vaccines, Vero cells are generally grown at ultra-high density. Exposure to chronic stress, nutrient depletion, metabolite accumulation, bursts of reactive oxygen species, and osmotic imbalance is known to activate autophagy and inhibit cell growth [14]. Thus, the delicate equilibrium of autophagy is critical for the survival and productivity of Vero cells. DDIT3, a target of the PERK, IRE1, and ATF6 pathways, is downstream and convergent, and has been implicated in the control of cell fate, yet its mode of action in Vero cells remains undefined. Our current data suggest that DDIT3 may play a role in orchestrating lysosomal function in addition to promoting autophagy in these cells.

When nutrients are deficient, cells initially try to adapt in order to survive. However, when nutrient deficiency becomes prolonged, these adaptive mechanisms fail, and cells die in an autophagy-dependent manner [15]. Earlier studies have shown that the limitation of oxygen and nutrients upregulates proteins of the ATG family and increases both autophagic flux and cell death [16]. We validated starvation-induced autophagy in Vero cells by determining the lipidated LC3-II/I ratio and monitoring p62 turnover by Western blotting. TEM revealed double-membrane autophagosomes. Consistent with our observations, Hao et al. reported that prolonged starvation in Hank’s balanced salt solution led to elevated levels of ULK1, Beclin-1, and LC3 in mouse embryonic fibroblasts (MEFs), indicating a time-dependent intensification of autophagy [17].

It should be noted that EBSS represents an acute and complete starvation model, which differs from the gradual nutrient depletion occurring in high-density suspension cultures. Nevertheless, this model is suitable for initial mechanistic exploration, as it rapidly and synchronously induces nutritional stress while excluding confounding fac-tors such as culture volume and the accumulation of metabolic waste. Importantly, the core signaling events triggered by EBSS, including amino acid withdrawal, mTOR inactivation, ER stress, and DDIT3 upregulation, are conserved during the metabolic stress caused by nutrient depletion in high-density suspension cultures. Moreover, validation experiments in suspension-adapted Vero cells confirmed that autophagy is significantly activated and DDIT3 expression is upregulated during the nutrient-depleted phase of high-density suspension culture. Consistently, the inhibition of autophagy, including targeting DDIT3, effectively improves cell density and viability under suspension conditions. Therefore, despite the inherent limitations of the acute EBSS model, the regulatory mechanisms identified are reproducible in the actual suspension culture system, providing a theoretical basis for optimizing Vero cell suspension culture processes by targeting DDIT3.

To identify starvation-responsive regulators of autophagy, we performed RT-qPCR and Western blotting. Of all differentially expressed genes, the levels of *CCN2* and *DDIT3* increased steadily over starvation time. *CCN2* is a pleiotropic cytokine that modulates autophagy. In fibrotic diseases, when cells are under stress and *CCN2* is activated, autophagy is stimulated, resulting in less injury [18]. Similarly, the knockdown of *CCN2* represses autophagy and differentiation [19]. Stress-responsive DDIT3, which plays a role in regulating autophagy and apoptosis, is induced by DNA damage and other stressors [20]. Liu et al. showed that DDIT3 frees Beclin-1 from the inhibitory Bcl-2 complex to activate autophagy in HCC cells [21]. The increased level of the pro-apoptotic protein DDIT3 after starvation indicates that this protein is activated under conditions of starvation. The expression of DDIT3 is positively correlated with autophagic activity in this system.

As the site of autophagic degradation, lysosomes participate in the fusion of autophagosomes, where multiple hydrolases work together to degrade and clear their contents. Li et al. were the first to show that an upstream UPR transcription factor, ATF4, drives lysosomal acidification during drug-induced autophagy in HEK293 cells [22]. Our analysis indicated that depletion of the DDIT3 gene prevented lysosomal proteolytic capacity and cathepsin B activity, as determined by Western blotting and fluorogenic substrate assays. Hence, the DDIT3 gene controls lysosomal fitness under stressful conditions.

ER_S_ is a self-rescue mechanism that is triggered by dysfunction of the ER within cells. The accumulation of unfolded or misfolded proteins, abnormal Ca^2+^ homeostasis, or oxidative insults can all act as powerful triggers. Cells activate the UPR in response to ER stress by three canonical sensors: PERK, IRE1, and ATF6 [23]. *DDIT3* is upregulated by the PERK pathway among downstream effectors and functions as a regulatory hub that integrates stress cues into autophagy and survival decisions. Previous studies have demonstrated that *DDIT3* binds to the SIRT1 promoter and activates its transcription, while SIRT1 further enhances autophagic flux via AKT signaling [24]. However, the precise mechanism by which *DDIT3* connects ER stress to autophagy in Vero cells remained unclear. In order to investigate the regulation of *DDIT3* expression and autophagy, we used 4-phenylbutyrate (4-PBA) to pharmacologically inhibit ERs in Vero cells. According to the data, *DDIT3* appears to be primarily controlled by ERs signaling under the conditions tested, and its induction seems to play an important role in promoting autophagy.

The ER is the largest reservoir of intracellular Ca^2+^, and its luminal Ca^2+^ is maintained for protein folding and organelle function. Changes in ER calcium (ER Ca^2+^) stores occur when a cell is subjected to stressful environmental or physiological conditions. On the ER membrane, IP3Rs function as major Ca^2+^ release channels, and their hyperactivation causes excessive Ca^2+^ efflux into the cytosol to activate PERK-IRE1α signaling [25]. By utilizing Ca^2+^-sensitive fluorescent probes, we found that the induction of starvation significantly increased both the cytosolic Ca^2+^ fluorescent signal and induced the ERs markers BIP and DDIT3. The use of 4-PBA caused a reduction in intracellular Ca^2+^ and a marked reduction in the expression of BIP and DDIT3. In line with our current findings, Ma et al. reported that 4-PBA alleviated PM2.5-induced ERs in mouse lung tissue by normalizing intracellular Ca^2+^, reducing reactive oxygen species (ROS), reducing apoptosis, and downregulating GRP78, ATF6, and DDIT3 [26]. Similarly, Liu et al. showed that microcystin induced ERs, enhanced the levels of ATF6, IRE1, and DDIT3, and increased the cytosolic concentration of Ca^2+^ [27]. 4-PBA treatment and DDIT3 knockdown were effective in reducing these ERs responses and preventing microcystin cytotoxicity. Our data suggest that starvation-induced DDIT3 expression in Vero cells is tightly regulated by ERs, supporting that DDIT3 is a critical link between ER stress and autophagy as well as cell fate.

**Figure 8:**
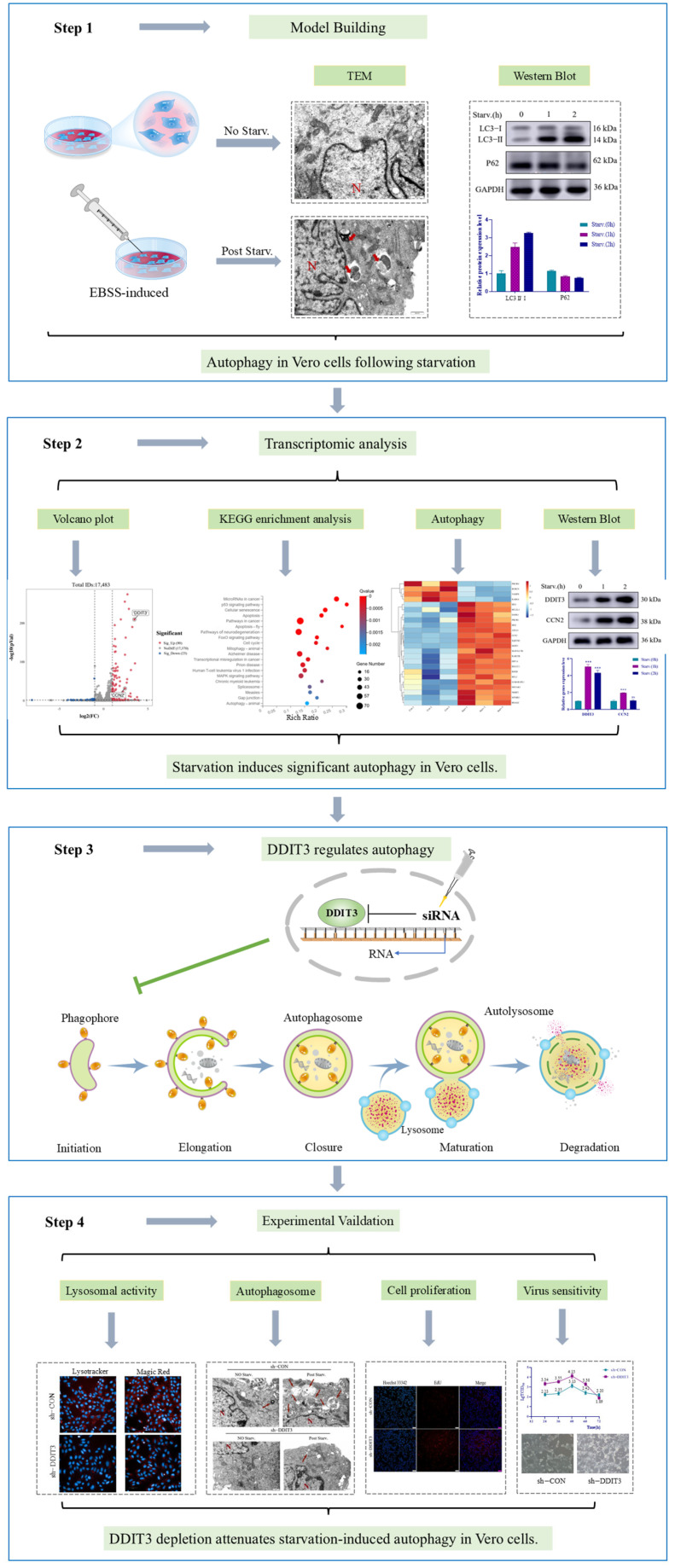
Schematic summary of DDIT3-mediated regulation of starvation-induced autophagy in Vero cells. This figure integrates morphological, transcriptomic, and functional evidence to elucidate the role of DDIT3 in autophagy regulation. Under EBSS-induced starvation, Vero cells exhibit time-dependent increases in LC3-II and decreases in p62 expression, confirming autophagy induction (Step 1). Transcriptomic analysis combined with KEGG enrichment identifies DDIT3 as a key autophagy-related gene upregulated upon starvation (Step 2). Functional validation demonstrates that DDIT3 depletion attenuates starvation-induced autophagy, as evidenced by reduced lysosomal activity, altered autophagosome formation, decreased cell proliferation, and enhanced virus sensitivity (Step 4). Collectively, this figure supports the conclusion that DDIT3 positively regulates starvation-induced autophagy in Vero cells (red arrows indicate autophagy bodies; N indicates the cell nucleus, *** *p* < 0.001).

In conclusion, this study provides evidence that DDIT3 may act as a starvation-inducible autophagy driver in Vero cells and may also play a role in regulating lysosomal competence. Our findings suggest that ER stress controls DDIT3 expression and, as a result, influences autophagic flux under the conditions tested. These data provide preliminary mechanistic insight into the DDIT3–ERs axis for cellular adaptation (Figure 8). While further validation is required, these findings suggest that DDIT3 may represent a promising molecular target to enhance the tolerance of Vero cells to nutrient deprivation and for the rational design of robust, suspension-adapted, genetically modified cell lines. It is noteworthy that DDIT3 knockdown attenuating autophagy differs from previous reports showing that DDIT3 silencing enhanced autophagy. This discrepancy likely reflects con-text-dependent functions of DDIT3. In previous studies, enhanced autophagy upon DDIT3 knockdown was observed in cancer cells treated with classical ERS inducers (e.g., tunicamycin), in which DDIT3 acted as a pro-apoptotic factor, and its downregulation indirectly restored autophagic flux. In contrast, in the present study, we applied nutrient starvation (EBSS), a metabolic form of stress that does not directly disrupt ER homeostasis. Under this condition, moderate DDIT3 upregulation may play an adaptive role, limiting excessive autophagy via the AKT/mTOR pathway. Thus, the role of DDIT3 in autophagy depends on stress type, cell lineage, and experimental timing, and our study reveals a pro-autophagic role for DDIT3 under starvation in Vero cells. However, several limitations of this study should be acknowledged. The evidence supporting DDIT3 regulation of the AKT/mTOR pathway is currently largely correlative: we observed increased p-AKT and p-mTOR levels upon DDIT3 knockdown, with no significant change in p-AMPK. The effects of DDIT3 overexpression on this pathway, as well as intervention experiments using pathway-specific agonists/inhibitors, have not yet been performed. We plan to address these issues in follow-up studies by carrying out the above experiments to confirm the causal relationship between DDIT3 and the AKT/mTOR pathway, as well as conducting ChIP-seq to identify downstream transcriptional targets of DDIT3 that may mediate this regulation.

## 4. Materials and Methods

### 4.1. Chemicals

TRIzol, an Evo M-MLV reverse transcription pre-mixed reagent kit, and an RT-qPCR reagent kit were purchased from Hunan Aikrui Biotechnology Co., Ltd. (Changsha, China); a BCA Protein Quantification Kit, RIPA lysis buffer, protease inhibitor, and 5× SDS-PAGE protein loading buffer were purchased from Beijing Solarbio Science & Technology Co., Ltd. (Beijing, China); Rabbit-derived LC3 antibody, rabbit-derived p62 antibody, rabbit-derived DDIT3 antibody, rabbit-derived CCN2 antibody, rabbit-derived TFEB antibody, rabbit-derived LAMP1 antibody, rabbit-derived BIP antibody, mouse-derived β-actin antibody, mouse-derived GAPDH antibody, horseradish peroxidase (HRP)-labeled goat anti-rabbit IgG, and HRP-labeled goat anti-mouse IgG were purchased from Hangzhou Huaan Biotechnology Co., Ltd (Hangzhou, China).

### 4.2. Cell and Virus Culture

sh-CON and sh-DDIT3 Vero cells with optimal growth conditions and no contamination were selected. Once cultures were fully confluent, we detached the cells with trypsin and resuspended the cells in an M199 medium supplemented with 5% NBS. Next, we seeded 20,000 cells per well in a 24-well plate and incubated these at 37 °C under 5% CO_2_. Each day, we collected and counted the number of cells from three replicate wells. Then, we created growth curves from the density data and determined the population-doubling time (*T*) and specific growth rate (*μ*) for each group, as shown below.


(1) $$T=\frac{t}{A}\;\;\;A={log}_{2}\frac{Y}{X}$$

The variables in the equation are defined as follows: *X*: initial cell count, *Y*: cell count recorded 24 h before the peak growth rate; *t*: elapsed culture time.

(2) $$\mu =({ln}^{X_{n}\cdot X_{n-1}})/(t_{n}-t_{n-1})$$

(*X*: density of viable cells; *t*: culture time; *n* and *n* − 1: two approached times).

When logarithmic growth was accomplished, the sh-CON and sh-DDIT3 monolayers were washed with PBS and infected with an influenza A/H1N1 virus at a multiplicity of infection (MOI) of 0.1 in a medium containing 1 µg mL^−1^ of TPCK-trypsin. The cytopathic effect (CPE) was examined at specific intervals of 1 day (24 h), 1.5 days (36 h), 2 days (48 h), 2.5 days (60 h), and 3 days (72 h) post-infection (pi). During this time course study, supernatant (SN) from the infected cells was harvested and stored at −80 °C for viral titer (TCID50 assay) determination.

### 4.3. Lentivirus Production and Cell Infection

The lentivirus that expresses short hairpin RNA (shRNA) that targets the sequence of the *DDIT3* gene GCAGCGCATGAAGGAGAAAGA, and that of a negative control, TTCTCCGAACGTGTCACGT, was synthesized and cloned into the GV493 (pFU-GW-016) vector with BsmBI sites (purchased from Shanghai Genechem Co., Ltd., Shanghai, China). Subsequently, DNA sequencing was used to detect the recombinant vector.

Green monkey DDIT3 (XM_008003778.2) was obtained from the cDNA library of Genechem (Shanghai, China) with the following primers: DDIT3 forward: 5′-GATCTATTTCCGGTGAATTCCGCCACCatggagctcgttccagccactcc-3′; and reverse: 5′-TCCTTGTAGTCCATGGATCCtgcttggtgcagattcaccattc-3′. The lentivirus vector plasmid GV828(CMV-MCS-3FLAG-EF1a-mCherry-T2A-neomycin) (purchased from Shanghai Genechem Co., Ltd., Shanghai, China), the vector, and the DDIT3 gene sequence were digested by AgeI and NheI restriction enzymes, and complete cloning was carried out using the In-fusion recombination method. The recombinant vector was detected by DNA sequencing.

Lipofectamine 2000 (Invitrogen; Thermo Fisher Scientific, Inc., Waltham, MA, USA) was used to transfect the viral vector into 293T cells along with two helper plasmids, psPAX2 and pMD2.G. Infectious lentiviruses were collected 72 h after transfection, and cell debris was removed by rapid centrifugation and filtered using 0.45 μm cellulose acetate filters. The analysis of GFP-positive 293T cells through FACS revealed the virus titer to be about 1 × 10^9^ transducing units (TU)/mL medium.

### 4.4. RNA Isolation, Library Preparation, RNA Sequencing, and Differentially Expressed Gene Analysis

TRIzol was used to isolate total RNA from Vero cells under starvation conditions (Starv-1, Starv-2, Starv-3) and control conditions (Con-1, Con-2, Con-3). The samples were then sent to BGI for sequencing after quality control. Poly-A mRNA was captured with oligo-dT magnetic beads and reverse-transcribed using random N6 primers, and the resulting cDNA was subjected to end repair, adaptor ligation, and PCR amplification to generate strand-specific libraries that were sequenced on the DNBSEQ platform.

Initially, all raw reads of low quality, high N, and supporting adapters were filtered to obtain clean reads. These were then aligned to the reference genome to predict novel transcripts and detect alternative splicing. Potential protein-coding transcripts were combined with the reference gene set to create a reference for gene expression quantification.

We utilized R for the analysis of transcriptomics data from BGI to assess differentially expressed mRNAs between the Con and Starv groups. We generated a volcano plot to display differentially expressed genes (DEGs) on the qPCR plate, with *p* < 0.05. Using KEGG annotation, the assigned genes and their biological function(s) were assigned to a biological pathway. Furthermore, the phyper function (*q* < 0.05, FDR-corrected) was used to evaluate the significance of enrichment.

### 4.5. Real-Time Quantitative PCR Assay

Gene-specific primers were designed based on the sequences of the target gene (Table 1). Total RNA was extracted using TRIzol reagent and quantified using a micro-volume spectrophotometer. Samples with A260/A280 ratios of between 1.9 and 2.1 were included in this study.

Reverse transcription took place in two stages. In the first step, 10 µL of genomic DNA removal reaction was performed by adding 2 µL of 4× gDNA wiper Mix and 1 µg of total RNA; then, RNase-free water was added to 10 µL. The reaction was then heated to 42 °C for 2 min. To the 20 µL RT reaction, we added 4 µL of 5× Evo M-MLV RT Reaction Mix; this was then adjusted to 20 µL with RNase-free water; this was then heated to 37 °C for 15 min and then 85 °C for 5 s. qPCR was performed using a 20 µL system containing 10 µL of 2× SYBR Green Pro Taq HS Premix, 2 µL of cDNA, 1 µL of each primer, and 6 µL of RNase-free water. The conditions for cycling involved pre-denaturation at 95 °C for 30 s followed by 40 cycles of 95 °C for 5 s and 60 °C for 30 s.

### 4.6. Protein Extraction and Western Blot Analysis

Cells were washed once with ice-cold PBS prior to lysis with RIPA buffer containing protease inhibitors. The BCA Protein Quantification Kit was used for protein determination. Samples were then mixed at a ratio of 4:1 with 5× SDS-PAGE loading buffer and boiled at 100 °C for 10 min. Proteins were then separated by 12% SDS-PAGE at 80 V (stacking) and 120 V (separation). Separated proteins were then transferred onto PVDF membranes at 75 V for 90 min. The membranes were blocked in TBST with 5% non-fat dried milk for 2.5 h at room temperature. Then, membranes were incubated overnight at 4 °C with primary antibodies (all diluted in 5% BSA-TBST): LC3, p62, CCN2, TFEB, and BIP (1:2000); DDIT3 (1:5000); LAMP1 (1:1000). The following morning, membranes were washed three times in TBST (5 min per wash) and then incubated with appropriate HRP-conjugated secondary antibodies (1:5000 for a period of 70 min at room temperature). The ECL chemiluminescence system was used to visualize bands, and quantification was performed with ImageJ-win64 (version 1.54).

### 4.7. Transmission Electron Microscopy (TEM)

To investigate autophagosome accumulation, cells were exposed to 200 ng mL^−1^ of bafilomycin A1 in EBSS for 1 h and 2 h. Monolayers were treated with ice-cold 2.5% glutaraldehyde at 4 °C for 2 h. Then, cells were dehydrated in graded concentrations of acetone (30, 50, 70, 80, 90, 95, and 100%; 10 min per step). Samples were then infiltrated with acetone/Epon-812 mixtures (3:1, 1:1, and 1:3; 30 min each) and finally embedded in pure Epon-812. Ultrathin sections (60–90 nm sections) were prepared on an ultramicrotome and collected on 200-mesh copper grids. Sections were then treated with 2% uranyl acetate for 10 min, followed by lead citrate for 5 min. Sections were then imaged on grids with a transmission electron microscope (JEOL Ltd., Tokyo, Japan), and autophagic vacuoles were investigated at ×25,000 magnification.

### 4.8. LysoTracker Red and Magic Red Staining

For lysosomal staining, sh-CON and sh-*DDIT3* Vero cells were plated in 96-well plates. LysoTracker Red was diluted to 1:10,000 in EBSS and added to each well before incubation at 37 °C for 1 h. After removing the dye, Hoechst 33342 (1:1000 in PBS, Thermo Fisher Scientific, Waltham, MA, USA) was used to stain the nuclei for 30 min at 37 °C. Images were captured on an HCLCI system (Thermo Fisher Scientific, Waltham, MA, USA).

For cathepsin B activity, sh-CON and sh-*DDIT3* cells were seeded as above. Magic Red stock (250×) was diluted 1:200 in EBSS. A volume of 50 µL was reconstituted in DMSO and incubated with samples for 1 h at 37 °C. After removing the dye from the cells, we stained the nuclei with Hoechst 33342 (1:1 000 in PBS, 30 min at 37 °C, RT) and imaged the plates on the same high-content platform (Thermo Fisher Scientific, Waltham, MA, USA).

### 4.9. Calcium Assay

The Fluo-4 AM stock was diluted to 5 µM and added to the cells, which were incubated at 37 °C for 30 min to load the probe, washed with PBS, and then incubated for another 20 min so that Fluo-4 AM was completely de-esterified inside the cells to Fluo-4. Flow cytometry was used to measure Fluo-4 fluorescence and analyze intracellular Ca^2+^.

### 4.10. Cycle Assay

Following EBSS treatment for two hours, the cell suspension was transferred to centrifuge tubes and centrifuged at 1000 rpm for 5 min. A working solution of propidium iodide (PI) was prepared in PBS at a concentration of 50 µg/mL. Following centrifugation, the pellet was resuspended in PI + RNase A; this was then incubated at 37 °C in the dark for 30 min. Finally, the cells were analyzed using flow cytometry.

### 4.11. Cell Proliferation Assay

sh-CON and sh-*DDIT3* Vero cells were plated in 24-well plates. The stock solution of 10 mM EdU was diluted in serum-free M199 to a concentration of 20 µM (1:500), and an equal volume was added to each well to give a final concentration of 10 µM. The cells were incubated for 24 h. Following labeling, the cells were fixed with 500 µL of 4% paraformaldehyde for 30 min at room temperature, then permeabilized with 0.5% Triton X-100 for 10 min at room temperature. The Click reaction was performed by adding the reaction mix and incubating at room temperature in the dark for 30 min. Using Hoechst 33342, the nuclei were counterstained (1:1000) for 30 min at room temperature in the dark and imaged using fluorescence microscopy (Carl Zeiss, Oberkochen, Germany).

### 4.12. TCID50 Assay

sh-CON and sh-*DDIT3* Vero cells were seeded in a 96-well plate. Then serial dilutions of the virus stock were prepared in M199 without serum. For each dilution, 100 µL was added to six replicate wells; wells were also included for the negative control with just 100 µL of medium. After 2 h of absorption at room temperature, 100 µL of complete M199 medium (supplemented with 5% NBS) was added in a 5% CO_2_ atmosphere. After three days in the incubator, the cytopathic effect was scored, and lgTCID50/mL was calculated using the Reed-Muench method to check the efficacy of the sample.

Proportionate distance = (percentage positive above 50% − 50%)/(percentage positive above 50% − percentage positive below 50%)(3)

lgTCID_50_ = (proportionate distance × log_10_ dilution factor) + log_10_ dilution of the highest dilution showing >50% CPE (4)

### 4.13. Statistical Analysis

All experiments were performed independently at least three times (*n* = 3). Data are presented as the mean ± standard deviation (SD). Statistical analyses were performed using GraphPad Prism 9.0 software (GraphPad Software, San Diego, CA, USA). Comparisons between two groups were analyzed using two-tailed Student’s *t*-test. Comparisons among three or more groups were analyzed using one-way analysis of variance (ANOVA) followed by Tukey’s post hoc test for multiple comparisons. A *p*-value < 0.05 was considered statistically significant. Significance levels are indicated as * *p* < 0.05, ** *p* < 0.01, and *** *p* < 0.001.

## Figures and Tables

**Table 1:** Primers used in this study.

Gene Name	Primer Sequence (5′ → 3′)	TM (°C)	MW (g/mol)
*DDIT3*	F: CACTGGCTTGGCTGACTGAGG R: TTCCGTTTCCTGGTTCTCCCTTG	64.4 63.9	6418.2 7028.6
*CCN2*	F: CTGTGAGGAGTGGGTGTGTGAC R: ACCAGGCAGTTGGCTCTAATCATAG	66.0 63.2	6850.5 7294.9
*BIP*	F: ATCAGGGCAACCGCATCA R: CGTCAAAGACCGTGTTCTCG	62.5 62.9	5507.6 6129.0
*GAPDH*	F: TGCCAAATACGATGACATCAAGAAGG R: TGTCGCTGTTGAAGTCAGAGGAG	68.1 66.8	7698.0 7098.6

## Data Availability

The original contributions presented in the study are included in the article. further inquiries can be directed to the corresponding author.

## Abbreviations

The following abbreviations are used in this manuscript:

BIP	Binding immunoglobulin protein
Baf.A1	Bafilomycin A1
DDIT3	DNA damage inducible transcript 3
MOI	Multiplicity of infection
TCID_50_	50% tissue culture infective dose
4-PBA	4-Phenylbutyric acid

## Appendix A. Results of Differential Genes Screening

**Table A1:** Expression levels of differentially expressed genes.

Gene Symbol	Relative Abundance						log2FC	*p*-Values
	Con-1	Con-1	Con-1	Starv-1	Starv-1	Starv-1		
*IRS2*	8.51	7.13	8.25	19.99	19.26	18.16	1.2594076280	0.0000000000
*CCN2*	10.64	11.39	10.83	27.61	25.82	31.43	2.419748245	0.0000000000
*NRBF2*	27.19	23.87	25.33	34.09	31.11	31.71	0.334555991	0.0064087787
*IRS1*	2.73	2.62	2.27	4.71	4.08	3.85	0.757317718	0.0000000269
*GABARAPL1*	70.79	61.07	60.69	84.62	79.43	84.26	0.399973117	0.0000000844
*DAPK1*	19.3	18.79	19.31	23.11	22.11	21.04	0.21357044	0.0136865273
*PIK3R1*	24.59	24.11	26.42	30.84	29.87	28.53	0.259635827	0.0001480126
*MTMR3*	8.24	7.94	8.35	10.46	9.63	9.91	0.286026487	0.0022072767
*RRAGC*	27.51	25.93	26.39	33.79	30.64	32.7	0.273410066	0.0083555746
*RHEB*	487.45	410.04	441.12	529.22	516.89	516.64	0.158582203	0.0142665032
*ATG14*	7.44	7.33	7.6	11	11.05	10.63	0.54049581	0.0000006250
*HIF1A*	221.4	202.63	223.85	260.8	251.6	254.61	0.238367428	0.0000037804
*MAP1LC3B*	65.35	62.03	65.25	72.96	77.68	74.05	0.213112031	0.0033668122
*RAB33B*	6.68	5.58	6.84	9.35	9.51	8.93	0.532157825	0.0054085963
*RB1CC1*	26.32	24.28	27.23	33.96	33.15	33.21	0.360646207	0.0000000136
*EIF2AK3*	15.98	13.76	13.97	18.86	18.97	18.77	0.36678873	0.0002731263
*BCL2*	58.14	48.89	55.49	62.86	65.54	62.15	0.221202336	0.0031510506
*SQSTM1*	458.59	467.24	459.28	684.51	662.32	671.21	0.537805196	0.0000000000
*BCL2L1*	157.83	168.82	151.25	189.68	184.25	183.55	0.216477386	0.0005742087
*DDIT3*	45.06	40.73	44.57	470.33	508.65	533.56	3.484987966	0.0000000000
*VAMP8*	114.17	119.57	115.52	101.06	96.77	96.77	−0.282067046	0.0265819246
*RAB8A*	52.42	57.92	55.19	46.44	46.32	44.57	−0.280680886	0.0009064476
*PIK3R4*	29.05	26.34	28.51	24.15	24.19	24.36	−0.212765913	0.0139075326
*RUBCN*	9.52	9.61	10.36	8.54	8.09	8.16	−0.255768904	0.0385323772
